# HIF-1α promotes the migration and invasion of hepatocellular carcinoma cells via the IL-8–NF-κB axis

**DOI:** 10.1186/s11658-018-0077-1

**Published:** 2018-05-31

**Authors:** Wenming Feng, Tao Xue, Sanxiong Huang, Qilin Shi, Chengwu Tang, Ge Cui, Guanghui Yang, Hui Gong, Huihui Guo

**Affiliations:** 1 Department of Hepatobiliary Pancreatic Surgery, The First People’s Hospital of Huzhou, No. 158 Guangchanghou Road, Huzhou, Zhejiang Province 313000 People’s Republic of China; 2 Central Laboratory, The First People’s Hospital of Huzhou, No. 158 Guangchanghou Road, Huzhou, Zhejiang Province 313000 People’s Republic of China; 3 Department of General Surgery, The First People’s Hospital of Huzhou, No. 158 Guangchanghou Road, Huzhou, Zhejiang Province 313000 People’s Republic of China; 4 Department of Pathology, The First People’s Hospital of Huzhou, No. 158 Guangchanghou Road, Huzhou, Zhejiang Province 313000 People’s Republic of China; 5 Department of Pharmacy, The First People’s Hospital of Huzhou, No. 158 Guangchanghou Road, Huzhou, Zhejiang Province 313000 People’s Republic of China

**Keywords:** Hepatocellular carcinoma, Hypoxia, HIF-1α, IL-8, NF-κB

## Abstract

**Background:**

Hypoxia plays a critical role in many cancers. Hypoxia inducible factor-1α (HIF-1α) is an important mediator of the hypoxia response. It regulates the expression of various chemokines involved in tumor growth, angiogenesis and metastasis but the associated pathway needs further investigation.

**Methods:**

The expression level of HIF-1α was determined in hepatocellular carcinoma (HCC) cells. The correlation of interleukin-8 (IL-8) and HIF-1α was assessed by knocking down HIF-1α. These cells were also used to assess its influence on HCC cell migration and invasion was checked. Pyrrolidinedithiocarbamate (PDTC), an inhibitor of NF-κB, was used to confirm the associated signaling pathway.

**Results:**

HIF-1α was significantly expressed in HCC cells and found to promote HCC cell migration and invasion in an IL-8-dependent manner. NF-κB was confirmed to be involved in the process.

**Conclusions:**

HIF-1α promotes HCC cell migration and invasion by modulating IL-8 via the NF-κB pathway.

## Background

Hepatocellular carcinoma (HCC) is the third most common cause of cancer mortality worldwide [1, 2]. Although treatments including surgery, chemotherapy and radiotherapy have been applied alone and various combinations, the prognosis of HCC patients remains poor [3]. Furthermore, mainly due to the high incidence of recurrence and metastasis after surgical resection, the overall 5-year survival rate is very low [4, 5].

Hypoxia, which is a common characteristic of solid tumors, has a role on migration and invasion [6, 7]. It was recently reported that hypoxia promotes HCC progression and invasion by overexpressing or stabilizing hypoxia-inducible factor-1 (HIF-1) [6, 8, 9].

The HIF-1 heterodimer complex, which contains two subunits, HIF-1α and HIF-1β, functions as a key transcription factor under conditions of hypoxia [10–12]. HIF-1β is a constitutively expressed subunit of the heterodimer complex, whileHIF-1α is an oxygen-regulated subunit that determines the activity of the complex. It is found expressed in various solid tumors, including HCC [13–15].

HIF-1α binds to hypoxia-responsive elements to activate the transcription of target genes in association with metastasis, invasion and metabolism [16–19]. In cancer, it can regulate the expression of numerous cytokines, chemokines and their receptors, increasing tumor dissemination, proliferation, angiogenesis and survival [20–23]. Although HIF-1α has been detected inmany solid tumors, its regulatory mechanisms in different cancers still require investigation.

Recent study has proved that co-expression of interleukin-8 (IL-8), also known as C-X-C motif ligand 8 (CXCL8), and HIF-1α is associated with metastasis and poor prognosis in HCC [24]. Here, we investigated the impact of siRNA-mediated knockdown of HIF-1α on IL-8expression level under conditions ofhypoxia and found that it decreased. We also determined that exogenous expression of IL-8 could restore the migration and invasion of HCC attenuated by HIF-1α knockdown.

Nuclear factor κB (NF-κB) has been reported to be involved in the regulation of proliferation and invasiveness through its regulation of the expression of several genes involved in the cell cycle machinery [25–27]. Activation of the NF-κB–IL-8 axis is associated with the promotion of colorectal cancer cell proliferation and metastasis [28]. To assess the role of this axis in HCC migration and invasion, we employed pyrrolidinedithiocarbamate (PDTC), an inhibitor of the NF-κB pathway. We demonstrated that HIF-1α could promote the migration and invasion of HCC by modulating IL-8 expression via the NF-κB pathway.

## Methods

### Cell culture

The human HCC cell lines HepG2 and SMMC7721 and the normal liver cell line WRL68 were obtained from the Shanghai Institute of Biological Sciences of the Chinese Academy of Sciences. The cells were cultured in Dulbecco’s modified Eagle’s medium supplemented with 10% FBS (GIBCO-BRL), in humidified atmosphere of 5% CO_2_ and 95% air at 37 °C. For the hypoxia experiments, the cells were incubated in a humidified Hetomultig as incubator in 1% O_2_, 5% CO_2_ and 94% N_2_.

### RNA isolation, reverse transcription and quantitative real-time PCR

Total RNA was extracted from cell lines using the Trizol reagent (Invitrogen) according to the manufacturer’s protocol. Reverse transcription was performed using a PrimeScript RT reagent kit (TaKaRa). For quantitative real-time PCR, the obtained cDNA was amplified using SYBR Premix Ex Taq (TaKaRa). Glyceraldehydes-3-phosphate dehydrogenase (GAPDH) was used as a control and experiments were performed in triplicate. The sequences of primers were:

- HIF-1α sense, 5′-GAACGTCGAAAAGAAAAGTCTC-3′
- HIF-1α antisense, 5′-CCTTATCAAGATGCGAACTCACA-3′
- IL-8 sense, 5′-CAGCCTTCCTGATTTCTGC-3′
- IL-8 antisense, 5′-GGGTGGAAAGGTTTGGAGTA-3′
- GAPDH sense, 5′-TGACTTCAACAGCGACACCCA-3′
- GAPDH antisense, 5′-CACCCTGTTGCTGTAGCCAAA-3′

### Western blot analysis

Total cell lysates were subjected to 10% SDS-PAGE. The proteins were transferred to nitrocellulose filter membranes, which were blocked for 1 h in 5% non-fat dry milk. The membranes were incubated with primary antibodies at 4 °C overnight and then with secondary antibodies at room temperature for 2 h. GAPDH was used as a gel loading control and the experiments were performed in triplicate.

### Cell migration and invasion assays

For the migration assay, transwell chambers (Corning) with 8 μm pore size polycarbonate filter inserts for 24-well plates were used according to the manufacturer’s instructions. 1 × 10^5^cells were seeded onto the upper compartment in 200 μl DMEM with 0.1% FBS followed by placement into wells containing 500 μl complete medium in the lower chamber for 24 h at 37 °C. The cells on the upper surface of the membrane were removed and the cells attached to the lower surface of the membrane were fixed and stained with Giemsa stain. The number of cells was counted under a microscope.

Invasion was assayed using the same procedure as the migration assay, except that 70 μl of 1 mg/ml Matrigel (BD Biosciences) was added to the upper face of the membrane. Assays were repeated three times.

#### Small interfering RNA (siRNA) and transient transfection

siRHIF-1α generated with the sequence 5′-GUGAUGAAAGAAUUACGAAUTT-3′ (sense) and 5’-AUUCGGUAAUUCUUUCAUCACTT-3′ (antisense) was used to generate pSilencer3.1-HIF-1α as previously described [24]. The scrambled sequence 5′-UUCUCCGAACGUGUCACGUdTdT-3′, 5′-ACGUGACACGUUCGGAGAAdTdT-3’was produced. IL-8 siRNAs were purchased from Santa Cruz Biotechnology. According to the manufacturer’s instructions, siRNA transfection was performed using Lipofectamine 3000 (Invitrogen).

#### Statistical analysis

All data are presented as the means ± SD. Differences between the groups were tested for statistical significance using Student’s *t-*test or the χ^2^-test. *p* < 0.05 was considered statistically significant.

## Results

### HIF-1α has a high expression level in HCC under conditions of hypoxia

**Fig. 1:**
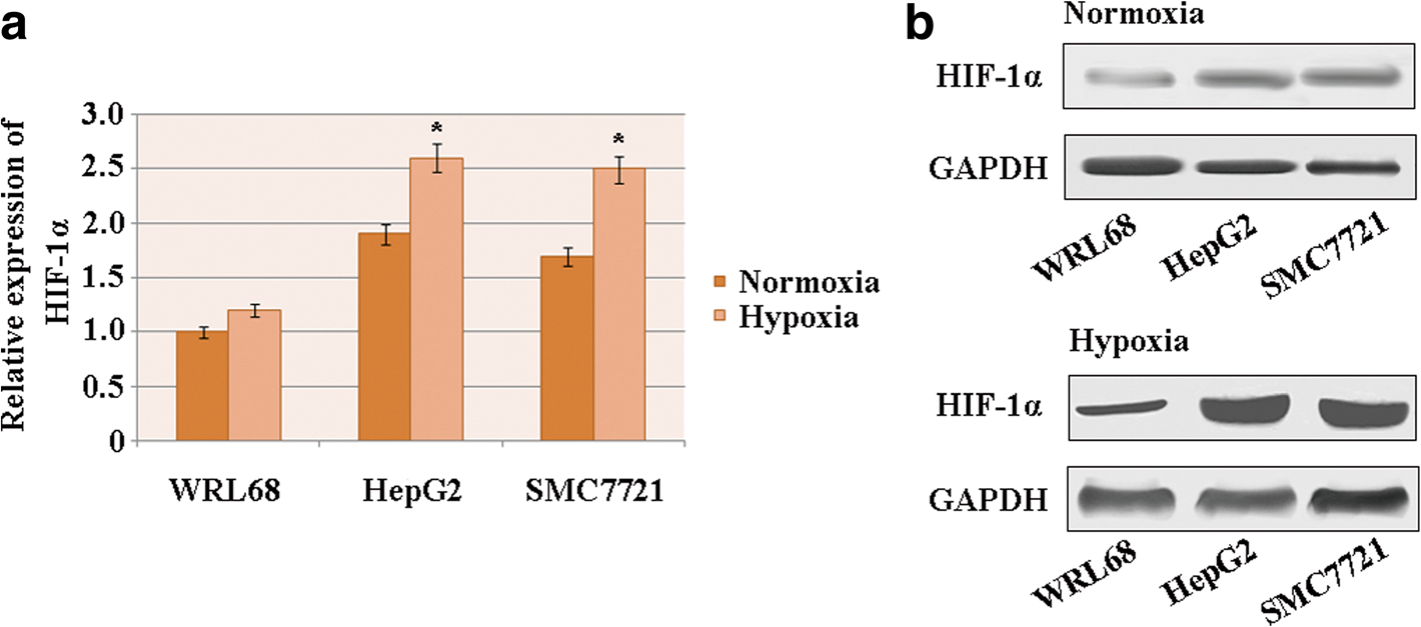
HIF-1α has a high expression level in HCC under conditions of hypoxia. **a** HIF-1α mRNA levels in human HCC cell lines and the normal liver cell line WRL68 (*n* = 3; *p < 0.05). **b** The expression of HIF-1α protein in HCC cell lines and the normal liver cell line WRL68. GAPDH was used as the loading control

The expression levels of HIF-1α were assessed via quantitative real-time PCR in the human HCC cell lines HepG2 and SMC7721 and compared to those for the normal liver cell line WRL68 under conditions of hypoxia and normoxia. The results show that under hypoxic conditions, HIF-1αexpression was higher in the HCC cell lines than in the normal liver cells. Similar observations were obtained under normoxic conditions (Fig. 1a).

Western blot analysis was performed to confirm the results. This showed remarkably higher expression levels of HIF-1α protein in HCC cell lines than in the normal liver cell line under hypoxic conditions (Fig. 1b), indicating that HIF-1α was induced in HCC cells by hypoxia.

### HIF-1α is associated with migration and invasion of HCC through regulation of IL-8 expression under hypoxic conditions

**Fig. 2:**
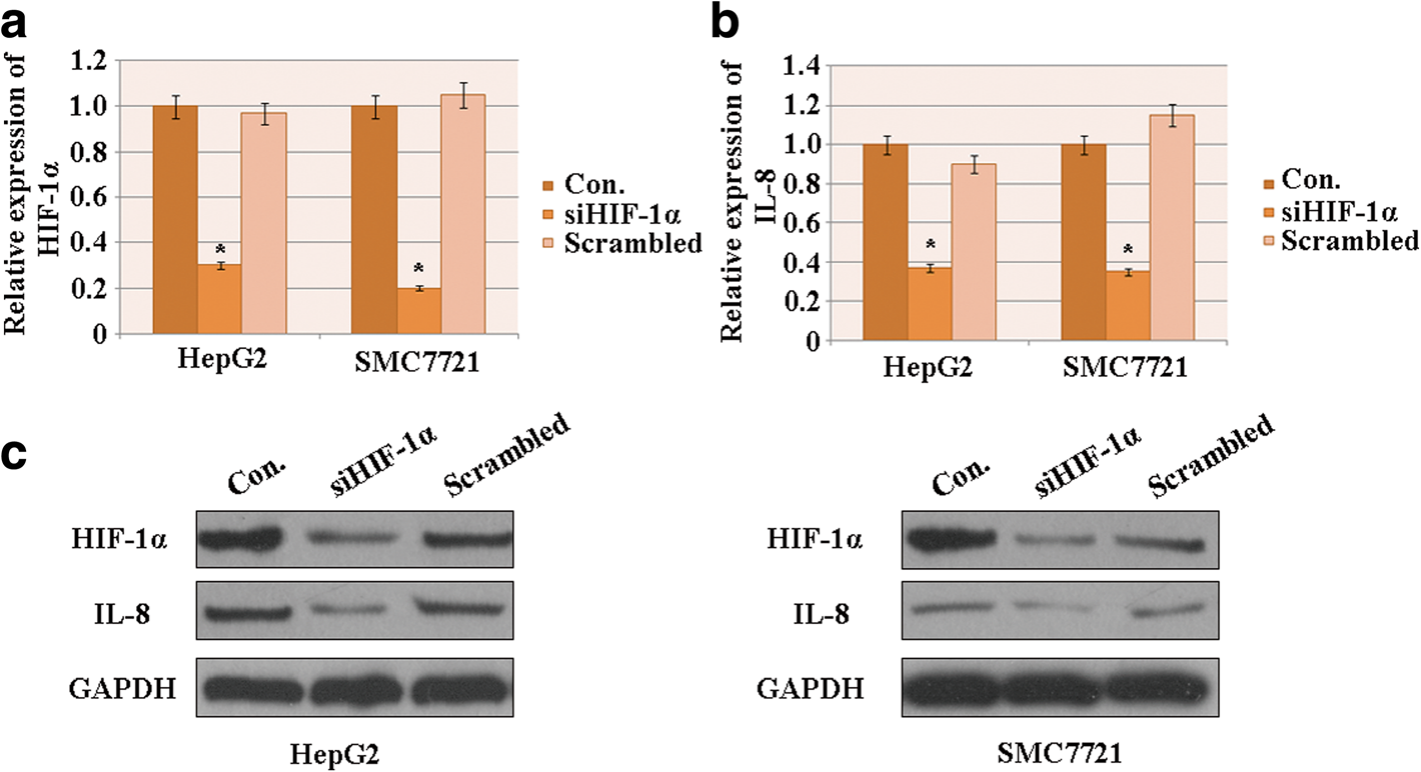
HIF-1α influences the migration and invasion of HCC by regulating IL-8 expression. **a** The efficiency of HIF-1α knockdown in HCC cell lines was evaluated using quantitative real-time PCR. **b** Quantitative real-time PCR analysis of IL-8 expression in response to HIF-1 knockdown in HCC cells. **c** Western blot analysis of HIF-1α and IL-8 expressions in HCC cell lines treated with siHIF-1α. GAPDH was used as control (*n* = 3; *p < 0.05). Con.: control

The efficiencyof siHIF-1α (the siRNA designed to knockdownHIF-1α) was evaluated via PCR and western blot assays for HCC cells under hypoxic conditions. siHIF-1α significantly decreased both the mRNA (Fig. 2a) and protein (Fig. 2c) levels of HIF-1α compared to the control and scrambled groups, indicating that siHIF-1α effectively silenced HIF-1α.

The effect of HIF-1α knockdown of IL-8 expression was investigated. Under hypoxic conditions, the expression levels of both IL-8 mRNA (Fig. 2b) and protein (Fig. 2c) markedly decreased after HIF-1α was knocked down. This suggests that under those conditions, the decrease inHIF-1αcorrelated with the downregulation of IL-8 expression in HCC cells.

**Fig. 3:**
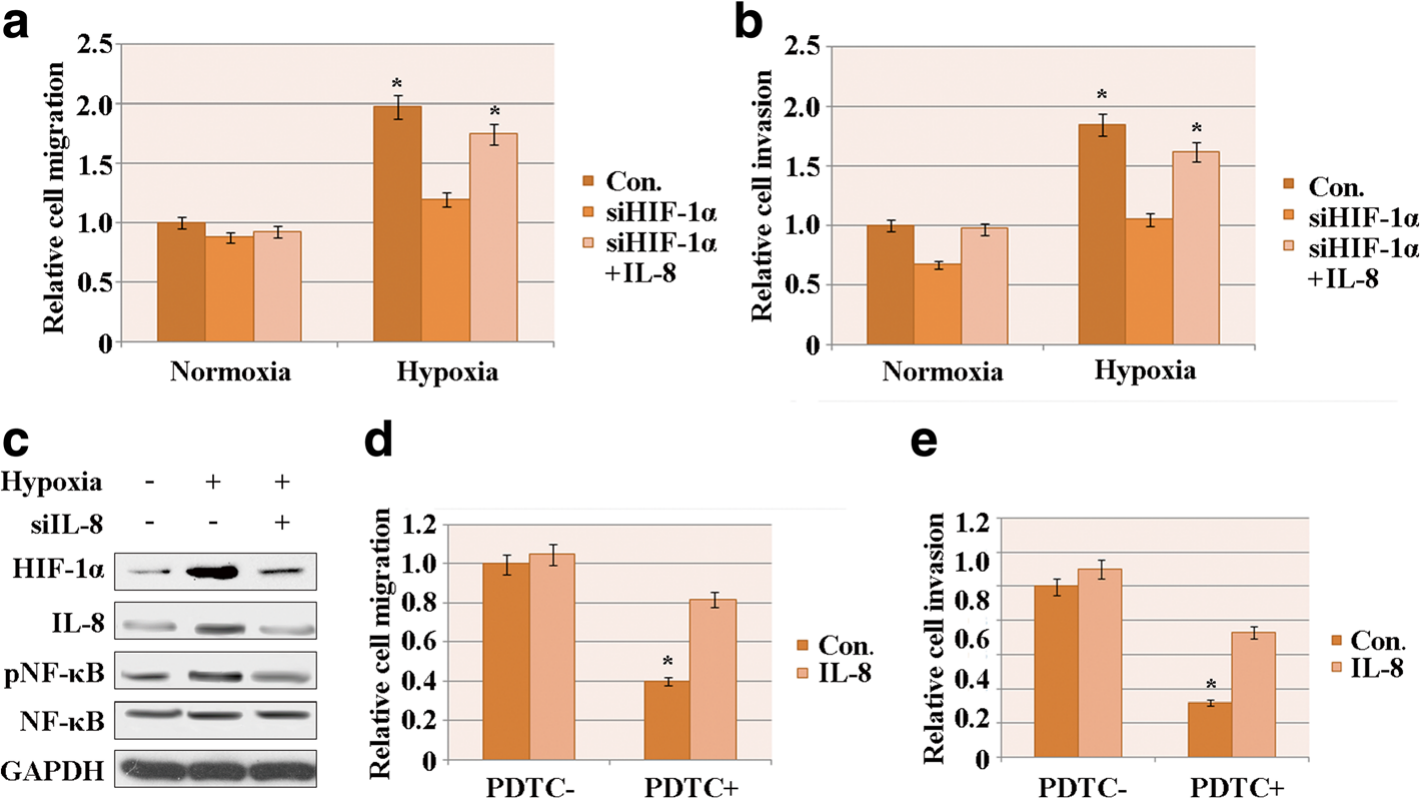
Overexpression of exogenous IL-8 restored the migration and invasion that had been attenuated by NF-κB pathway inhibition. **a** HepG2 cell migration aftersiHIF-1α treatment and subsequent IL-8 treatment under conditions of normoxia and hypoxia. **b** HepG2 cell invasion after siHIF-1α treatment and subsequent IL-8 treatment under conditions of normoxia and hypoxia. **c** Western blot assay of HIF-1α, IL-8, pNF-κB and NF-κB after siIL-8 treatment. GAPDH was used as the loading control. **d** HepG2 cell migration was decreased by PDTC when IL-8 was overexpressed. **e** HepG2 cell invasion was decreased by PDTC when IL-8 was overexpressed. The results represent three independent experiments. Con.: control; PDTC-: treated without PDTC; PDTC+: treated with PDTC

To investigate the effect of HIF-1α on HCC cell migration, transwell assays were performed in HepG2 cells under conditions of hypoxia and normoxia. According to the cell number analysis, cell migration was dramatically attenuated by knockdown of HIF-1α under hypoxic conditions. Under normoxic conditions, the relative cell migration did not decrease significantly due to knockdown of HIF-1α (Fig. 3a). However, exogenously overexpressing IL-8 increased the number of migrated cells relative to instances with knockdown of HIF-1α (Fig. 3a). Therefore, HIF-1α may affect HCC cell migration by regulating IL-8 expression under hypoxia but not under normoxia.

The invasive capacity of HepG2 cells was also evaluated using the transwell assay. The siHIF-1α had similar effects as in the migration assay (Fig. 3b), indicating that under hypoxic conditions, HIF-1α expression correlates and promotes with HCC cell invasion by regulating IL-8 expression.

### Overexpression of exogenous IL-8 restored the migration and invasion attenuated by NF-κB pathway inhibition

It was previously reported that the NF-κB–IL-8 axis is associated with metastasis of colorectal cancer cells [28]. To evaluate whether this axis is involved in HCC cell migration and invasion, and whether the correlation between HIF-1α and IL-8 expression levels is associated with the NF-κB pathway, IL-8 was knocked down.

The western blot assay was performed to the detect protein levels of HIF-1α and NF-κB. The IL-8 expression level was significantly decreased whensiIL-8 was transfected into HepG2 cells under hypoxic and normoxic condition, confirming the efficiency of siIL-8 (Fig. 3c). The protein levels of HIF-1α and p-NF-κB also decreased after IL-8 was knocked down in HCC cells under hypoxia (Fig. 3c), implying a correlation between HIF-1α, IL-8 and NF-κB.

We employed an NF-κB pathway inhibitor, PDTC, to assess the role of the NF-κB–IL-8 axis on HCC cell migration and invasion. Hep2G cells were treated with 30 μM PDTC, followed by the transwell assay to evaluate the migration and invasion of HCC cells under hypoxia. When treated with PDTC, the number of migrated and invaded HCC cells sharply decreased (Fig. 3d, e), suggesting that the inhibition of NF-κB attenuated HCC cell migration and invasion under hypoxia.

IL-8 was also overexpressed in HCC cells with or without PDTC treatment. Overexpressing IL-8 effectively enhanced theHCC cell migration and invasion that had been found to be attenuated by NF-κB inhibition. However, we did notfind a significant effect of IL-8 overexpression on cells without PDTC treatment. Therefore, IL-8 may affect HCC cell migration and invasion through the NF-κB pathway under conditions of hypoxia.

## Discussion

Hypoxia can promote metastasis and tumor angiogenesis by inducing HIF-1 [29–33]. The determinative subunit of HIF-1, HIF-1α, has a role in regulating hundreds of genes, including cytokines, chemokines and their receptors [20, 21, 34, 35]. Previous studies have reported that HIF-1α is upregulated in HCC cells. This is consistent with our results, which showthat both the mRNA and protein levels of HIF-1α were higher in HCC cells than in a normal liver cell line under conditions of hypoxia. This indicates that hypoxia promotes HIF-1α expression in HCC cells.

IL-8, also known as CXCL8, is an important regulator of metastatic and advanced cancers. It serves as an autocrine growth factor that promotes tumor growth, metastasis and angiogenesis [36, 37]. There is a reported correlation between serum IL-8 expression levels and tumor size and stage of HCC [38], and IL-8 is known to be involved in stimulating HCC cell invasion and metastasis [39]. Recent study has confirmed that IL-8 is associated with metastasis and poor prognosis in HCC when co-expressed with HIF-1α [24].

Here, we observed that HIF-1α expression correlates with the expression level of IL-8, as evidenced by the downregulation of IL-8 in response to silencing of HIF-1αin HCC cell lines under hypoxic conditions. Furthermore, IL-8 compensated for the attenuation of HCC cell migration and invasion caused by knockdown of HIF-1α. This suggests that the induction of HIF-1α by hypoxia promoted HCC cell migration and invasion in an IL-8-dependent manner.

NF-κB has been reported to be involved in the regulation of proliferation and invasiveness through its influence on the expression of several genes that are key to the cell cycle machinery [25–27]. Activation of the NF-κB–IL-8 axis is associated with the promotion of colorectal cancer cell proliferation and metastasis [28]. In our study, the inhibition of NF-κB by PDTC significantly attenuated HCC cell migration and invasion. Overexpression of exogenous IL-8 elevated the migratory and invasive capacities of HCC cells. Therefore, the NF-κB–IL-8 axis also has an important role in the regulation of HCC cell migration and invasion.

## Conclusions

HIF-1α and IL-8 are upregulated in HCC cells. Here, we demonstrated thatHIF-1α promotes HCC cell migration and invasion by modulating IL-8 via the NF-κB pathway. These results should be further verified in vivo to establish if targeting HIF-1α or IL-8 with siRNA has promise for human therapeutic application against HCC.

## Abbreviations

CXCL8: C-X-C motif ligand 8
HCC: Hepatocellular carcinoma
HIF-1α: Hypoxia inducible factor-1α
IL-8: Interleukin-8
NF-κB: Nuclear factor κB
PDTC: Pyrrolidinedithiocarbamate
